# Anthropometric Typology of Male and Female Rowers Using K-Means Clustering

**DOI:** 10.2478/v10078-011-0032-y

**Published:** 2011-07-04

**Authors:** Justyna Forjasz

**Affiliations:** 1 Department of Anatomy and Anthropology, Academy of Physical Education in Poznań, Faculty of Physical Education in Gorzów Wlkp

**Keywords:** rowers, somatic variables, anthropometric typology

## Abstract

The aim of this paper is to present the morphological features of rowers. The objective is to establish the type of body build best suited to the present requirements of this sports discipline through the determination of the most important morphological features in rowing with regard to the type of racing boat. The subjects of this study included competitors who practise rowing and were members of the Junior National Team. The considered variables included a group of 32 anthropometric measurements of body composition determined using the BIA method among male and female athletes, while also including rowing boat categories. In order to determine the analysed structures of male and female rowers, an observation analysis was taken into consideration and performed by the k-means clustering method. In the group of male and female rowers using long paddles, higher mean values for the analysed features were observed, with the exception of fat-free mass, and water content in both genders, and trunk length and horizontal reach in women who achieved higher means in the short-paddle group. On the men’s team, both groups differed significantly in body mass, longitudinal features, horizontal reach, hand width and body circumferences, while on the women’s, they differed in body mass, width and length of the chest, body circumferences and fat content. The method of grouping used in this paper confirmed morphological differences in the competitors with regard to the type of racing boat.

## Introduction

In light of the research performed, there is no doubt that rowing is one of those sport disciplines in which certain morphological characteristics of a competitor are a prerequisite condition to the achievement of high results. At the same time, the differentiation of body build increased depending on the type of rowing boat (short paddles, long paddles) and specific rowing events.

Research by Piotrowski et al. (1992), Skład et al. (1993), Bourgois et al. (2000), Stewart (2001), Claessens et al. (2005), Battista et al. (2007), Mikulić (2008), Alacid et al. (2011) and Forjasz (2011) indicated that rowers differ from non-athletes not only in body height and body weight, but also in the length of the upper and lower limbs (especially of the shank), width of the shoulders, width of the distal parts of the upperand lower limbs, and large muscular girths of the limbs (especially of the forearm). In light of the results of those authors, characteristics for competitive rowers include proper relationships between tissue components, along with low body fat content and high fat-free mass. Martirosow et al. (1985), on the other hand, considered body height, body weight, horizontal reach, trunk length, lower limb length, as well as muscle and fat mass the most diagnostic features in male and female rowers.

The analysis of model characteristic features for male and female junior rowers can be a way of gradual assessment of a competitors’ adaptation to particular training loads, and assessment of the preparation and development of young rowers, for whom the age of a junior is only a stage on the way to a sports championship.

The aim of this paper is to establish the morphological features of rowers. The authors tried to determine the type of body build best suited to the present requirements of this sports discipline through the determination of the most important morphological features in rowing with regard to the type of racing boat. The analysed type of body build is the result of selection for rowing and the realisation of specific training loads.

The results of the research are a theoretical consideration of the model body build for male and female rowers, while practical implications for coaches include criteria for selection.

## Material and methods

The subjects of this research included competitors who practise rowing and were members of the Junior National Team. Anthropometric measurements were performed in 2005. Anthropological measurements of 28 male rowers and 26 female rowers were performed (Table 1).

Male rowers and female rowers included in the research belong to the junior category, which includes competitors at the age of 16–18, with training experience ranging from 0,5 to 5 years (Table 2). During the research, the athletes were in the preparation period, in the sub-period of extensive preparation.

Analysis included direct and indirect measurements of somatic variables, taking into consideration those especially diagnostic in rowing (Martirosow et al., 1985; Bourgois at al. 2001; Mikulić, 2008; Alacid et al., 2011; Forjasz, 2011):

1. length features: body height, trunk length, upper limb length, arm length, forearm length, hand length, lower limb length, thigh length, shank length, and foot length,
2. width features: shoulder width, chest width, chest depth, pelvis width, foot width, and hand width,
3. body circumferences: chest circumference (at rest), waist circumference, abdomen circumference, hip circumference, arm circumference (at rest, at mid-length) forearm circumference (maximum), thigh circumference (maximum), and shank circumference (maximum),
4. body mass,
5. thickness of skin folds: under the shoulder blade, arm (triceps), forearm, abdomen, thigh and shank,
6. horizontal reach of upper limbs – the highest obtuse angle of upper limbs (distance of anthropometric points daIII of right and left hand with upper limbs spread out).

The measurements were taken according to anthropometric principles, with the use of classical instruments. Body mass was registered with an electronic scale (precision to 0,1kg); the length measurements were taken by anthropometer (0,1cm); width measurements, with a small and large bow compass (0,1cm); the circumferences were measured with an anthropometric tape measure (0,1cm); thickness of skin folds were marked with a pair of compasses with a calliper (0,2mm); and the horizontal reach of the upper limbs with an anthropometric tape measure (0,1cm).

Body composition was also evaluated. It was conducted by bioelectrical analysis of impedance, establishing the quantity of water, fat and lean body tissue (“Scectrum Lightweight II”).

The consent of the Bioethical Committee at the Poznan University of Medical Sciences (No. 1758/03), was obtained to perform the research project.

Survey data gave essential information on age and training experience of competitors (Table 2).

Collected material was analysed with basic statistical methods, listing measures of central tendency and variability: arithmetic mean (M) and standard deviation (SD) of individual features for all examined athletes with regard to the type of row boat (short paddles, long paddles). Minimum and maximum values were added to the list to show the range of measured variables. The significance of variation of mean features was estimated with the use of Student’s t-test for independent variables.

In order to determine the analysed structures of male and female rowers, the method of observation analysis was taken into consideration, and was performed by k-means clustering. K-means clustering is based on the verification of a hypothesis as to the number of observations among analysed cases (subjects). The calculative procedure starts with k-random chosen observations for the cases, which are then clustered by observation, with a minimisation of variability for each (StatSoft, 1997). In the analysis of observations, 32 somatic features and 3 body components were taken into account.

Cluster analysis aims to arrange objects in such a way that the degree of relation between objects belonging to the same group was as large as possible, and with objects from other groups as small as possible. The analysis of observations uncovers structures in the data without an explanation of their causes. To the types of methods of observation analysis belongs a method of agglomeration and grouping using k-means. Grouping by k-means consists of forming as few different observations as possible, while taking into consideration a minimization of variability for each observation and a maximization of variability between observations. Then, the maximum possibility will characterise the members of a given group and the rest of the objects.

## Results

The existence of two observations was predetermined. The observations corresponded with the number of racing boats (short paddles, long paddles). Analysis revealed the existence of two observations (Table 3):

- for the men’s team: first – the majority of male rowers using short paddles, second – the majority of male rowers using long paddles.
- for the women’s team: first – the majority of female rowers using short paddles, second – the majority of female rowers using long paddles.

The observations are presented by the following numbers:

- men: observation 1 – 12 subjects; observation 2 – 16 subjects,
- women: observation 1 – 15 subjects; observation 2 – 11 subjects.

**Figure 1:**
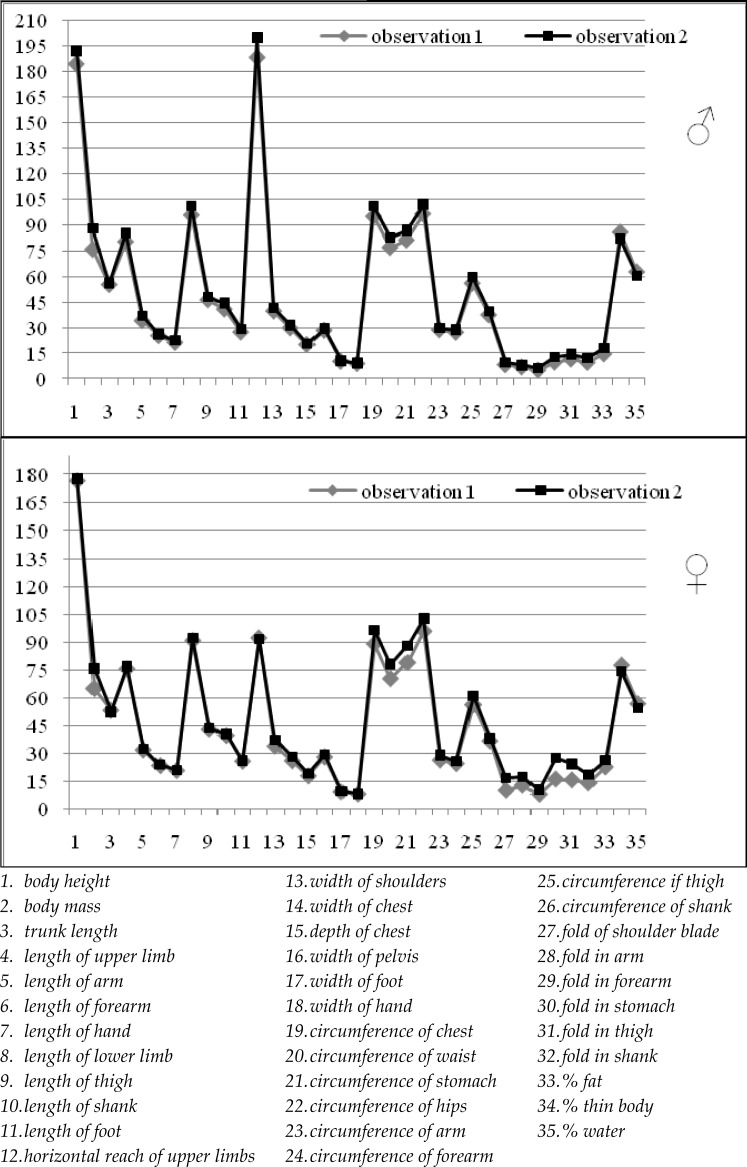
Graphical presentation of average values of somatic features and body components of men and women in particular observations

For both sexes two observations were separated: first by majority of short-paddle competitors and second by majority of long-paddle competitors. Separated observations can be estimated according to their level of somatic features. For both sexes, taking into account average values of morphological features, the second observation (i.e. the long-paddle group, figure 1), is classified at a higher level. In the group of male rowers using long paddles, larger average values of analysed features were observed, with the exception of percentage of fat-free mass and total water, which reached higher average values in the group using short paddles. For female rowers, higher average features for long-paddle competitors were observed, with only the trunk length, horizontal reach, percentage of fat-free mass and water content showing higher average values among women using boats with short paddles.

The following activity concentrates on establishing to what extent the applied criterion, that is the type of racing boat, is reflected in the morphological features of both groups‘ observations. The observations are characterised by many variations in analysed variables (Table 3).

Sex as a differentiating feature for both observations was accounted for by differences in: body mass, chest width, chest, waist, abdomen, hip, forearm, and thigh circumferences, skin fold thickness under the lower angle of shoulder blade, the arm, the forearm, and shank, as well as percentage of fat, fat-free mass and water content. Moreover, for the men’s groups of short paddles and long paddles there were significant differences in body height, upper limb, arm, hand, shank and foot length, horizontal reach, hand width and shank circumference. The analysed female groups presented significant differences in shoulder width, chest thickness, pelvis width, arm circumference, and skin fold thickness of the abdomen and thigh (Table 4–5).

## Discussion

The research established the dependence between specific body constitution and performance in rowing. This paper analysed the somatic features of competitors using short and long paddles. Rowing technique with the use of long paddles generally differs from that using of short paddles. Differences result from the use of one instead of two paddles by a rower, and the asymmetrical work of whole body. Beginners in this sports discipline begin rowing training with the use of short paddles, with consideration for health and prophylactic reasons (symmetrical work is more advisable for proper physical development of the young athletes), safety reasons (it is easier to keep the balance in the boat) and training benefits (by mastering rowing technique with a short paddle it is easier to master rowing with long paddles).

In an analysis of the achieved results, it was observed that both sexes had higher means of all analysed length features for the team using long paddles with the exception of forearm and thigh length (women). Higher means of all width variables, which were subject to research, were also observed in the group of competitors specialised in using long paddles. The male team participating in long-paddle competition was characterised by larger chest, abdomen, hip, forearm and shank circumference, and women achieved higher means for all analysed circumferences. Rowers using short paddles had a greater waist, arm and thigh circumferences. The analysis also showed general body mass, greater skin fold thickness and bigger horizontal reach in long-paddle competitors. Models of body constitution of competitors depending on the type of competition generally known from the literature (Skład et al., 1994; Bourgois et al., 1997, 2000; Fell & Gaffney, 2001; Claessens et al., 2002; Jürimäe & Jürimäe, 2002; Desgorces et al., 2004; Claessens et al., 2005; Slater et al., 2006; Forjasz & Kuchnio, 2009; Forjasz, 2011) are confirmed in the research on young rowers. However, the literature does not provide detailed information on the body build of competitors with divisions for different types of racing boats (long paddles, short paddles). Competitors in the junior category, with respect to their young age, are not limited to only one event, and the type of boat remains unchanged (information collected by means of interview with the coach of the Junior National Team). Therefore there are difficulties in verification of the achieved results. Proven trends in morphological characteristics were confirmed by Krupecki (2003) in his study, which indicated crucial differences between competitors using short and long paddles who took part in Olympic Games in Barcelona and Atlanta. It showed that the competitors using short paddles had shorter body height (by 2,6 cm) and lower body mass (by 2,8 kg) than athletes using long paddles. Differences observed in the groups were caused by selection criteria, optimization of the training process and coaches‘ increased knowledge in exercise physiology, biochemistry and anthropology. Body constitution is one of the indicators for selection to teams with respect to type of racing boat. Among other factors the following should also be mentioned: ability of kinaesthetic differentiation of movement (“feeling the boat”, “feeling the paddle”) and mental predispositions of the competitor (individual or group competition, ability to cooperate in a group). The number of associated competitors in a rowing club is also important. In clubs with a smaller number of rowers, short paddles are used more often because of a simpler team selection process, which requires fewer competitors and gives the possibility of individual starts in boats of the “single” type (information collected by interview with the coach of Junior National Team).

Analysis of somatic variables of male and female rowers revealed the morphological differences of competitors depending on the type of racing boat. A question was also posed to find whether the somatic structure can be used in the taxonomy of cases. Cluster analysis by k-means clustering method was performed. For both sexes, the existence of two observations was established: one with the majority of short-paddle competitors, and the other with the majority of long-paddle competitors. The cluster analysis expresses the differentiation of athletes with regard to type of rowing boat. For the group of rowers using long paddles, higher mean values of analysed features were observed, with the exception of percentage of fat-free mass and water content, which reached the highest values in the group using short paddles. For women, higher values were observed in long-paddle competitors; only trunk length, horizontal reach, percentage of fat-free mass and water content showed higher means in female rowers using short paddles. For the men’s team, both groups significantly differ in body mass, length features, horizontal reach, hand width, and body circumferences, and for the women, in body mass, length and width measurements of the chest, body circumferences and fat content.

In the overall view of the obtained results, it can be stated that rowers of Junior National Team are characterised by significant body height and body mass, while maintaining a low BMI. Characteristic features include a long trunk and large circumferences, long upper and lower limbs, broad shoulders, a narrow pelvis and a flat chest. High levels of musculature of the upper and lower limbs are also characteristic and they distinguish the competitors in the group with stocky limbs and high body adiposity, identified by skin fold thickness and percentage of fat content. The female group is characterised by a similar body build with narrow shoulders, a medium-wide pelvis, and medium arching of the chest. The analysed teams show differences dependent on the types of racing boat, with higher feature means among the long paddle users, with higher body fat content. Comparing the described body build of a rower to the one described by Mikołajczak (1970) we can state that within 40 years the biggest changes concern shoulder width, pelvis width and development of the chest. Those values are smaller in beginner rowers. The morphology of world-class paddlers appears to have altered during the past 25 years toward a more compact, robust physique, which is especially noticeable amongst female competitors (Ackland at al. 2003).

In the last few years a champion model often called the “model of leading functions” has become the model to which one strives, and which shows the direction of particular sport disciplines. It is regarded as a crucial element in improving the effectiveness of sports training. These elaborate models can be used during recruitment for particular sports disciplines, as well as in the selection of competitors and the evaluation of their further developmental possibilities. “The champion model” should take into account the competitor’s age, training experience, somatic build, level of directed fitness preparation and technical, tactical and mental preparation. Optimisation of activities with consideration of biological development and target rates is essential (Malina, 1994; Ackland & Bloomfield, 1996; Hawkins, 2000; Abbott & Collins, 2002; Beunen at al. 1992; Malina at al., 2004; Williams & Kendall, 2007; Vaeyens at al., 2008). Collecting data from different scientific fields, including anthropology, will highlight the mechanisms shaping fitness, find causes of often observed stagnation, state its temporary or permanent character, and formulate more precise developmental directions.

## Conclusion

The method of grouping cases used in this paper confirmed the morphological differentiation of the competitors depending on the type of racing boat. In the groups of male and female rowers using long paddles, higher mean values for the analysed variables were observed, with the exception of percentage of fat-free mass and water content, as well as trunk length and horizontal reach by women, who achieved higher means in the short-paddle group. In men, both groups differed significantly in body mass, longitudinal variables, horizontal reach, hand width, and body circumferences, while in women, in body mass, width and length measurements of the chest and body circumferences.

## Figures and Tables

**Table 1:** Number of examined teams of men and women

men			women		
total	short paddles	long paddles	total	short paddles	long paddles
28	10	18	26	11	15

**Table 2:** Age and training experience of male and female rowers (M-mean; SD - standard deviation)

	men				women			

	min	max	M	SD	min	max	M	SD
age (yrs)	15,97	17,77	17,08	0,51	15,53	17,83	16,98	0,70
years of training	1,17	5,50	3,13	1,41	0,50	5,00	2,80	1,34

**Table 3:** Separation of observation by k-means for the teams of men and women

**men**		**Women**	

**observation 1**	**observation 2**	**observation 1**	**observation 2**
sp (1)	sp (2)	sp (1)	sp (5)
sp (3)	sp (4)	sp (2)	sp (7)
sp (6)	sp (5)	sp (3)	lp (1)
sp (7)	lp (1)	sp (4)	lp (2)
sp (8)	lp (2)	sp (6)	lp (3)
sp (9)	lp (3)	sp (8)	lp (8)
sp (10)	lp (4)	sp (9)	lp (11)
lp (6)	lp (5)	sp (10)	lp (12)
lp (9)	lp (7)	sp (11)	lp (13)
lp (10)	lp (8)	lp (4)	lp (14)
lp (14)	lp (11)	lp (5)	lp (15)
lp (18)	lp (12)	lp (6)	
	lp (13)	lp (7)	
	lp (15)	lp (9)	
	lp (16)	lp (10)	
	lp (17)		

sp -short paddles (rower number)

lp - long paddles (rower number)

**Table 4:** Average somatic features and body components of men in determined observations

	**observation 1**		**observation 2**		

	M	SD	M	SD	t value
body height (cm)	184,47	3,83	191,99	4,21	4,684^**^
body mass (kg)	75,78	5,80	87,66	5,73	5,203^**^
trunk length (cm)	55,21	2,10	56,07	1,99	1,066
length of upper limb (cm)	80,28	2,00	85,32	2,81	5,095^**^
length of arm (cm)	33,95	1,65	36,77	1,56	4,447^**^
length of forearm (cm)	25,17	1,94	26,09	1,25	1,464
length of hand (cm)	21,17	1,04	22,47	1,60	2,371^*^
length of lower limb (cm)	95,61	3,48	100,85	4,20	3,382^**^
length of thigh (cm)	46,24	3,04	47,96	3,49	1,308
length of shank (cm)	40,80	2,12	44,33	2,39	3,912^**^
length of foot (cm)	27,27	0,99	29,01	1,11	4,156^**^
horizontal reach of upper limbs	188,32	4,21	199,86	4,73	6,449^**^
width of shoulders (cm)	39,64	2,73	41,47	2,06	1,943
width of chest (cm)	29,68	1,49	31,39	1,79	2,590^*^
depth of chest (cm)	19,98	1,15	20,64	1,04	1,530
width of pelvis (cm)	28,27	1,91	29,31	1,45	1,587
width of foot (cm)	10,07	0,64	10,43	0,55	1,514
width of hand (cm)	8,69	0,32	9,07	0,36	2,795^**^
circumference of chest (cm)	94,84	3,05	100,69	3,68	4,311^**^
circumference of waist (cm)	76,92	3,18	82,57	3,46	4,265^**^
circumference of stomach (cm)	81,25	3,80	86,79	4,32	3,405^**^
circumference of hips (cm)	96,34	2,87	101,86	3,90	3,982^**^
circumference of arm (cm)	28,59	1,42	29,76	1,76	1,811
circumference of forearm (cm)	27,13	1,25	28,64	1,17	3,142^**^
circumference if thigh (cm)	55,97	2,79	59,52	3,50	2,783^**^
circumference of shank (cm)	37,32	2,02	39,38	1,88	2,678^*^
fold of shoulder blade (mm)	8,02	1,53	9,65	1,90	2,356^*^
fold in arm (mm)	6,69	1,04	7,86	1,54	2,188^*^
fold in forearm (mm)	4,90	0,80	5,96	1,17	2,601^*^
fold in stomach (mm)	9,55	2,51	12,53	5,08	1,798
fold in thigh (mm)	11,37	3,50	14,29	4,37	1,833
fold in shank (mm)	9,40	3,01	12,20	3,06	2,324^*^
fat (%)	14,42	2,97	17,88	3,77	2,528^*^
thin body (%)	85,58	2,97	82,12	3,77	2,528^*^
water (%)	62,67	2,27	60,19	2,66	2,500^*^

* p<0,05

** p<0,01 for differences between concentration 1 and concentration 2

**Table 5:** Average somatic features and body components of women in determined observations

	**observation 1**		**observation 2**		

	M	SD	M	SD	t value
body height (cm)	176,31	2,79	177,67	6,05	0,737
body mass (kg)	64,77	4,89	75,67	4,36	5,646^**^
trunk length (cm)	53,08	2,53	52,39	3,86	0,528
length of upper limb (cm)	75,41	1,64	76,80	3,01	1,446
length of arm (cm)	31,63	1,32	32,10	2,25	0,636
length of forearm (cm)	23,22	0,89	23,75	1,31	1,190
length of hand (cm)	20,56	0,94	20,95	1,40	0,809
length of lower limb (cm)	90,66	2,66	92,22	4,25	1,102
length of thigh (cm)	42,83	2,60	43,65	2,52	0,765
length of shank (cm)	39,33	1,25	40,23	3,06	0,981
length of foot (cm)	25,57	0,75	25,88	0,80	0,991
horizontal reach of upper limbs	92,08	2,09	91,53	2,72	0,564
width of shoulders (cm)	33,67	3,67	37,21	3,26	2,447^*^
width of chest (cm)	25,80	1,99	28,04	1,42	3,055^**^
depth of chest (cm)	17,69	0,63	19,15	1,18	3,915^**^
width of pelvis (cm)	27,85	1,26	29,26	1,24	2,736^*^
width of foot (cm)	9,08	0,51	9,35	0,40	1,420
width of hand (cm)	7,81	0,30	7,96	0,35	1,192
circumference of chest (cm)	88,87	3,34	96,28	4,92	4,393^**^
circumference of waist (cm)	70,13	2,61	78,04	3,99	5,867^**^
circumference of stomach (cm)	78,81	3,62	87,92	4,23	5,673^**^
circumference of hips (cm)	95,71	2,85	102,64	1,91	6,723^**^
circumference of arm (cm)	26,22	1,59	28,89	1,85	3,794^**^
circumference of forearm (cm)	24,27	0,86	25,61	1,40	2,885^**^
circumference if thigh (cm)	56,09	2,67	60,97	2,10	4,831^**^
circumference of shank (cm)	36,43	1,96	37,93	2,67	1,581
fold of shoulder blade (mm)	9,91	1,42	16,75	5,91	4,148^**^
fold in arm (mm)	12,73	2,68	17,31	3,70	3,509^**^
fold in forearm (mm)	7,71	1,87	10,25	2,58	2,806^**^
fold in stomach (mm)	16,03	5,16	27,29	6,27	4,819^**^
fold in thigh (mm)	15,55	4,88	24,15	6,91	3,574^**^
fold in shank (mm)	13,96	3,48	18,58	4,64	2,791^**^
fat (%)	22,47	2,20	26,27	3,80	3,089^**^
thin body (%)	77,40	3,36	74,09	2,47	2,659^*^
water (%)	56,60	2,38	54,27	1,90	2,568^*^

* p<0,05

** p<0,01 for differences between concentration 1 and concentration 2
